# Elastic Graphene‐Shell Architectures Enable Stress Regulation in High‐Capacity Lithium‐Ion Battery Electrodes

**DOI:** 10.1002/advs.78085

**Published:** 2026-10-20

**Authors:** Luwen Li, Qitao Shi, Zhipeng Wang, Cheng Zhang, Jiaqi Wang, Xiaoqin Yang, Alicja Bachmatiuk, Chen Lu, Ruizhi Yang, Yanbin Shen, Mark H. Rümmeli

**Affiliations:** ^1^ College of Energy Soochow Institute for Energy and Materials Innovations (SIEMIS) Key Laboratory of Advanced Carbon Materials and Wearable Energy Technologies of Jiangsu Province Soochow University Suzhou P. R. China; ^2^ Suzhou Laboratory Suzhou China; ^3^ Faculty of Chemistry Wrocław University of Science and Technology Wrocław Poland; ^4^ Electron Beam Emergent Additive Manufacturing (EBEAM) Centre Centre For Nanotechnology (CNT) Centre For Energy and Environmental Technologies (CEET) VSB—Technical University of Ostrava Ostrava Czech Republic; ^5^ School of Electronic and Information Engineering Changshu Institute of Technology Changshu China; ^6^ i‐Lab CAS Center for Excellence in Nanoscience Suzhou Institute of Nano‐Tech and Nano‐Bionics (SINANO) Chinese Academy of Sciences (CAS) Suzhou P. R. China; ^7^ IFW Institute For Materials Chemistry Dresden Germany; ^8^ School of Smart Materials and Future Energy Anhui Normal University Wuhu China

**Keywords:** graphene‐shell spheres, pore‐engineering additives, silicon‐based anodes, stress regulation

## Abstract

Silicon‐based anodes are limited by large volume changes, stress accumulation, and sluggish kinetics during cycling. Porous electrode designs can alleviate these problems, but conventional approaches often rely on multistep processing or sacrificial pore formers. Here, hollow graphene‐shell (GS) spheres are introduced as slurry‐compatible pore‐engineering additives. In a 5–60–5 MPa pressure cycle, the SiO_x_/GS electrode shows greater thickness recovery than pristine SiO_x_, with thickness‐recovery indices of 4.18 and 1.24 percentage points, respectively. Individual GS particles show a largely recoverable response within the small‐displacement range probed by atomic force microscopy (AFM), with an apparent modulus of approximately 114 MPa. Upon full lithiation, the through‐plane thickness of the SiO_x_/GS electrode increases by 15%, compared with 94% for pristine SiO_x_. The SiO_x_/GS electrode also retains approximately 702 mAh g^−1^ after 400 cycles at 1.6 A g^−1^. GS improves the performance of the tested SiO_x_/graphite, Si@C, and LiNi_0.8_Co_0.1_Mn_0.1_O_2_(NCM811) formulations as well. These results demonstrate a simple, slurry‐compatible strategy for accommodating electrode deformation while maintaining electrochemical performance.

## Graphical abstract

In this study, we developed elastic hollow graphene spheres (GS) that serve as a pore‐engineering additive. This strategy enables the direct fabrication of porous electrodes, effectively mitigating volume expansion and mechanical stress in silicon‐based anodes during cycling without compromising active material or increasing production costs. The GS‐modified SiO_x_ electrode exhibits superior mechanical resilience and cycling stability, delivering 701.9 mAh g^−1^ after 400 cycles at 1.6 A g^−1^, with broad applicability to other high‐capacity electrode materials.

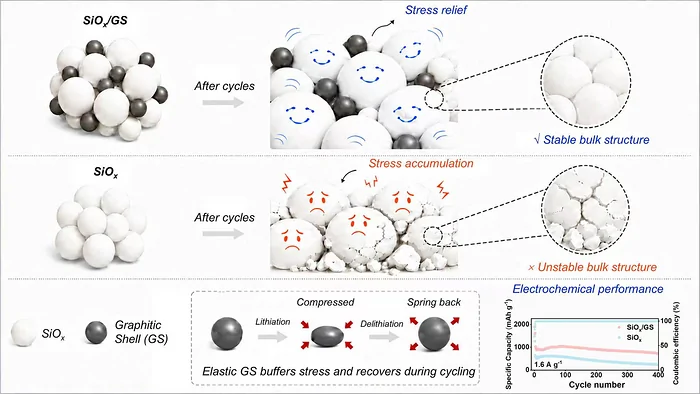

## 1 Introduction

The demand for high‐energy‐density lithium‐ion batteries (LIBs) for electric vehicles and portable electronics has spotlighted Si‐based anodes owing to their high theoretical capacity [1, 2, 3]. However, their use is hindered by large particle volume changes during cycling [4], which can result in particle pulverization, loss of electrical contact, and capacity underutilization [5, 6, 7]. To overcome these limitations, porous electrode designs [8, 9], such as porous Si structures [10, 11] and yolk–shell architectures [12], have been explored [13, 14, 15, 16]. These designs are typically realized through conventional porosity‐engineering methods, such as chemical etching [17], thermal treatment [18], and the use of pore‐forming agents such as NH_4_HCO_3_ [19], inorganic salts like NaCl [20], polymethyl methacrylate [21], and (NH_4_)_2_SO_4_ [22]. However, these methodologies still face several challenges that limit their implementation [23, 24, 25].

Specifically, chemical etching often leads to excessive material removal owing to compositional anisotropy, resulting in active material loss and capacity degradation. The excessive surface area promotes side reactions and reduces the volumetric energy density (Figure S1). Similarly, thermal treatment methods rely on volatile precursors, and the resulting pore size and distribution are highly sensitive to processing conditions [26, 27, 28]. Uneven pore structures can lead to inconsistent ion transport and lithiation, thereby deteriorating battery performance (Figure S2). The introduction of pore‐forming agents results in additional issues, such as residual agents forming an organic‐rich solid electrolyte interphase (SEI) that impedes Li^+^ transport or thermal decomposition reactions generating gaseous byproducts, causing battery swelling and safety concerns (Figure S3). More critically, conventionally fabricated porous electrodes suffer from pore‐structure collapse and progressive densification during cycling, which undermines their original design intent [29, 30].

A central challenge in Si‐based anode design is to create porous structures that accommodate stress while maintaining transport and structural continuity. Here, graphene‐shell (GS) spheres are used as slurry‐compatible pore‐engineering additives. Their hollow dimensions are defined by the precursor SiO_2_ template, and the internal voids are introduced directly during slurry processing. Within the small‐displacement range examined by AFM, individual particles show a largely recoverable local response with an apparent modulus of approximately 114 MPa, while post‐compression SEM shows that their spherical morphology is retained after treatment at 200 MPa. These features support the proposed stress‐accommodating role of GS, together with possible contributions from the conductive shell and porous architecture to electronic transport and electrolyte access.

Using SiO_x_ as the active material, the SiO_x_/GS electrode shows a smaller through‐plane thickness increase, improved capacity retention, and a higher relative LiF contribution after cycling under matched conditions. GS addition also improves performance in the tested SiO_x_/graphite, Si@C, and NCM811 formulations. These findings support a multifunctional pore‐engineering strategy that combines mechanical accommodation with transport and interfacial effects.

## 2 Results and Discussion

### 2.1 Characterization of GS and GS‐Modified Electrodes

The GS spheres were fabricated by chemical vapor deposition (CVD) using SiO_2_ spheres as sacrificial templates and acetonitrile as the precursor [31, 32]. After deposition, the template was removed by etching with 5 mol L^−1^ NaOH, yielding a hollow structure (Figure S4). SEM images show uniform spherical particles with an average diameter of approximately 1 µm (Figure S5). TEM reveals a three‐dimensional hollow structure bounded by thin graphitic layers (Figure 1d). EDS mapping shows a homogeneous distribution of C, N, and O across the sphere surfaces (Figures 1e–e_3_), consistent with the XPS results (Figure 1f). C and N originate from acetonitrile decomposition during CVD, whereas the O signal is mainly attributed to surface oxidation upon air exposure [33]. The XRD pattern contains the graphitic (002) reflection at 26.6° (Figure S6). The Raman I_D_/I_G_ ratio of 0.95 indicates a defect‐rich graphitic framework (Figure S7), which may provide additional electrochemically accessible sites [34]. The type‐IV N_2_ adsorption–desorption isotherm (Figure S8) indicates mesoporosity on the GS surface [31].

**FIGURE 1:**
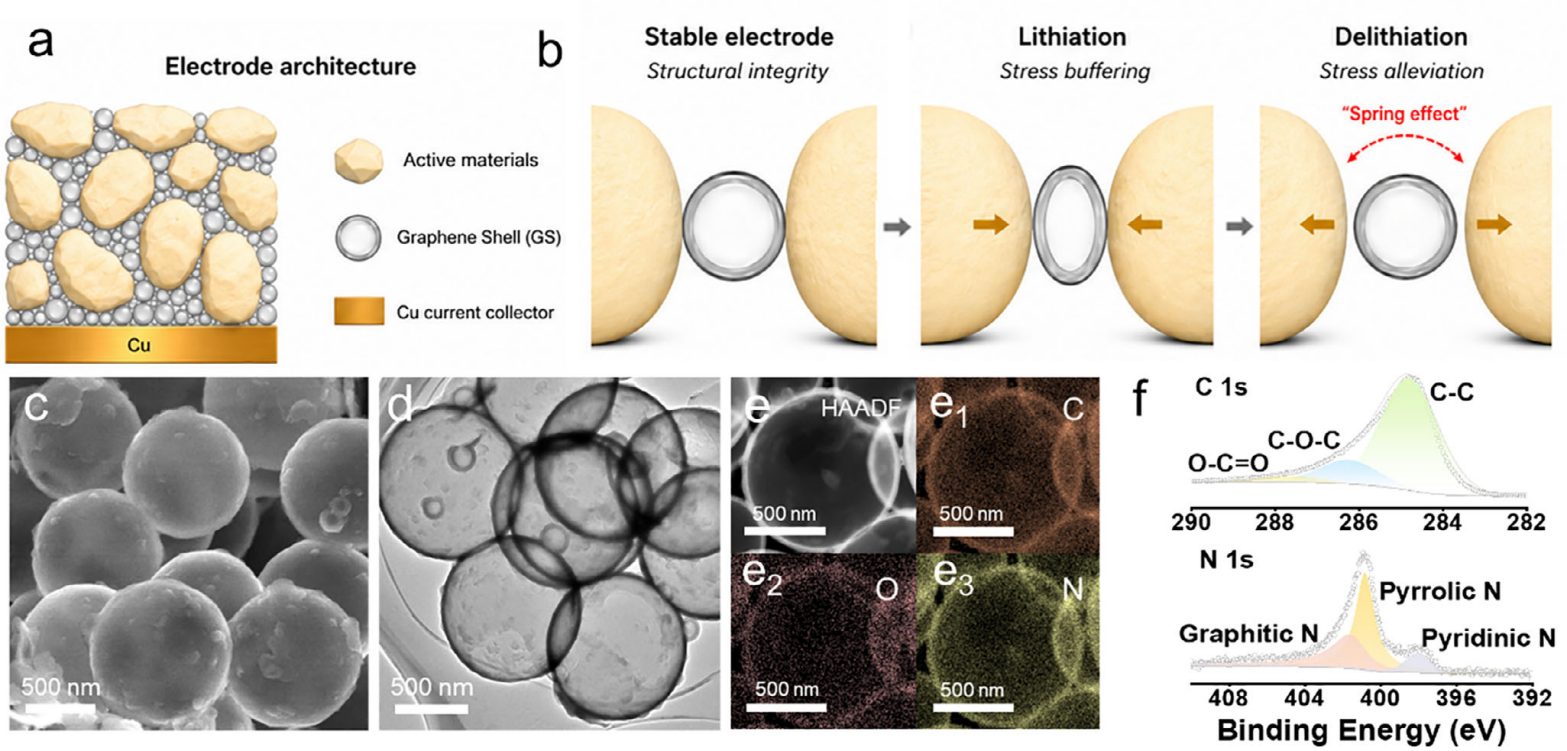
(a) Schematic of porous electrode incorporating GS spheres. (b) Mechanism of stress alleviation by GS spheres during lithiation. During lithiation, the expanding active material compresses the elastic spheres, which deform under pressure; upon delithiation, the spheres recover their structure, preserving structural integrity.(c) SEM, (d) TEM, and e HAADF images of GS, (e1–e3) EDS elemental mapping of GS. (f) High‐resolution XPS spectra of C 1s and N 1s.

To visualize the pores introduced by hollow GS particles, a 20 wt,% GS electrode was prepared solely for cross‐sectional imaging by nondestructive argon‐ion milling. The milled particles appear as concave hemispheres distributed among the SiO_x_ particles (Figure 2a), confirming that GS introduces hollow space into the composite. AFM force spectroscopy was then used to probe the local mechanical response of an individual GS particle (Figure 2b). The approach and withdrawal traces nearly overlap over an approximately 0.01 µm displacement range (Figure 2c), indicating limited hysteresis and a largely recoverable response within this small‐displacement regime [35]. Hertz fitting yielded an apparent whole‐particle modulus of approximately 114 MPa. In a separate powder measurement, the conductivity increased from approximately 0.5 to 2.8 S cm^−1^ as pressure rose from 0 to 200 MPa (Figure 2d). Post‐compression SEM images showed that the particles remained largely spherical without obvious shell fracture after this single high‐pressure treatment.

**FIGURE 2:**
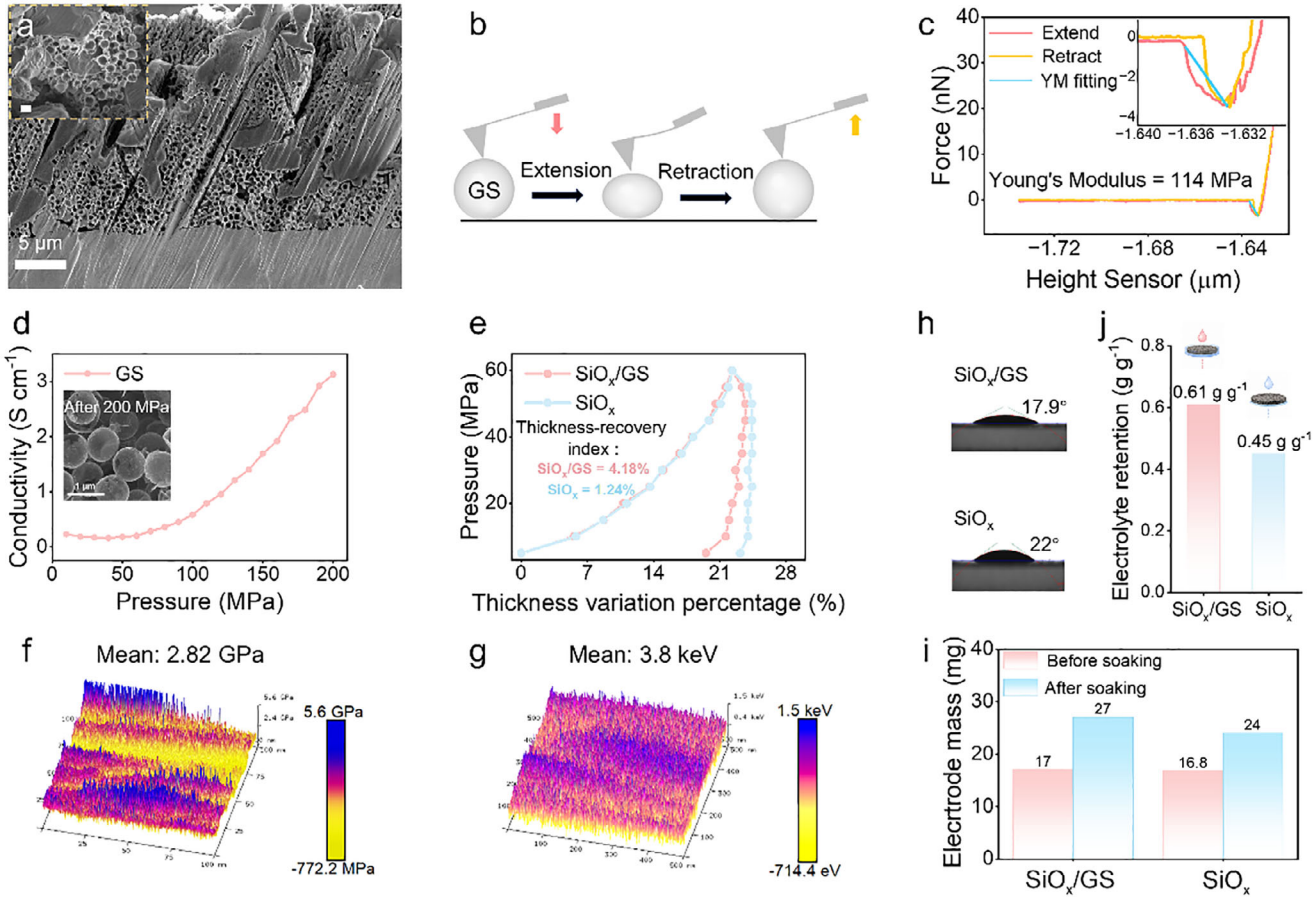
(a) Cross‐sectional FESEM images of a 20 wt,% GS electrode prepared solely for pore visualization. Scale bar in the inset: 2 µm. (b) Schematic of the AFM force measurement. (c) Force–displacement traces of an individual GS particle over the tested small‐displacement range. (d) Pressure‐dependent conductivity of GS powder; inset: SEM image after compression at 200 MPa. (e) Percentage reduction in thickness of the optimized SiO_x_/GS and pristine SiO_x_ electrodes during a 5–60–5 MPa pressure cycle. (f) Apparent Young's modulus distribution of the fresh SiO_x_/GS electrode. (g) Energy‐dissipation distribution of the fresh SiO_x_/GS electrode (map mean: 3.8 keV). (h) Electrolyte contact angles. (i, j) Electrolyte‐retention measurements. The AFM means and distributions in f,g describe the displayed maps.

GS contents of 2, 4, and 6 wt,% were screened before the electrode‐level mechanical comparison; the formulations are listed in Tables S1 and S2, and the electrochemical, morphological, and impedance results identified 4 wt,% as the optimal formulation tested (Figures S9–S12). During a 5–60–5 MPa pressure cycle, the optimized SiO_x_/GS and pristine SiO_x_ electrodes showed comparable loading responses, but the GS‐containing electrode recovered more thickness during unloading (Figure 2e). We quantified this response using the thickness‐recovery index, I_rec_ = ε_max_ − ε_final,5 MPa_, where ε_max_ is the maximum recorded thickness reduction and ε_final,5 MPa_ is the residual reduction at the final 5 MPa point. The resulting indices were 4.18 percentage points for SiO_x_/GS and 1.24 percentage points for pristine SiO_x_ (Tables S3 and S4); relative to ε_max_, the corresponding recovery fractions were 17.6% and 5.1%. Because the maximum reduction occurred shortly after pressure reversal, this operational index captures both elastic recovery and time‐dependent relaxation. The GS‐containing electrode also maintained higher conductivity throughout the pressure sequence (Figure S13) [36]. In the AFM maps of the fresh electrodes, the mean apparent modulus was lower for SiO_x_/GS (2.82 GPa) than for pristine SiO_x_ (45.7 GPa; Figure 2f and Figure S14), and the mean energy dissipation was 3.8 vs. 6.3 keV (Figure 2g and Figure S15). These values are means over the displayed maps, not averages over independent electrodes [37, 38] The measured contact angles were 17.9° for SiO_x_/GS and 22.0° for SiO_x_, while the electrolyte‐retention values were 0.61 and 0.45 g g^−1^, respectively (Figure 2h–j). These wetting and retention results are consistent with improved electrolyte accessibility in the GS‐containing electrode [39].

### 2.2 Electrochemical Performance and Kinetics

The electrochemical behavior of the SiO_x_/GS electrode was first evaluated by cyclic voltammetry (CV) at 0.2 mV s^−1^ (details of battery fabrication are provided in the Supporting Information) [40]. As shown in Figure 3a, a distinct cathodic peak appeared at 1.78 V (vs. Li^+^/Li) during the first cycle, which can be assigned to the formation of the solid electrolyte interphase (SEI) derived from fluoroethylene carbonate decomposition [41]. The disappearance of this peak in subsequent cycles indicates the formation of a stable interphase. Meanwhile, the gradually increasing current response of the SiO_x_/GS electrode suggests an activation process and improved reaction kinetics within the electrode [33]. In contrast, the pristine SiO_x_ electrode shows a much weaker current response, which is associated with its higher internal resistance (Figure S16).

**FIGURE 3:**
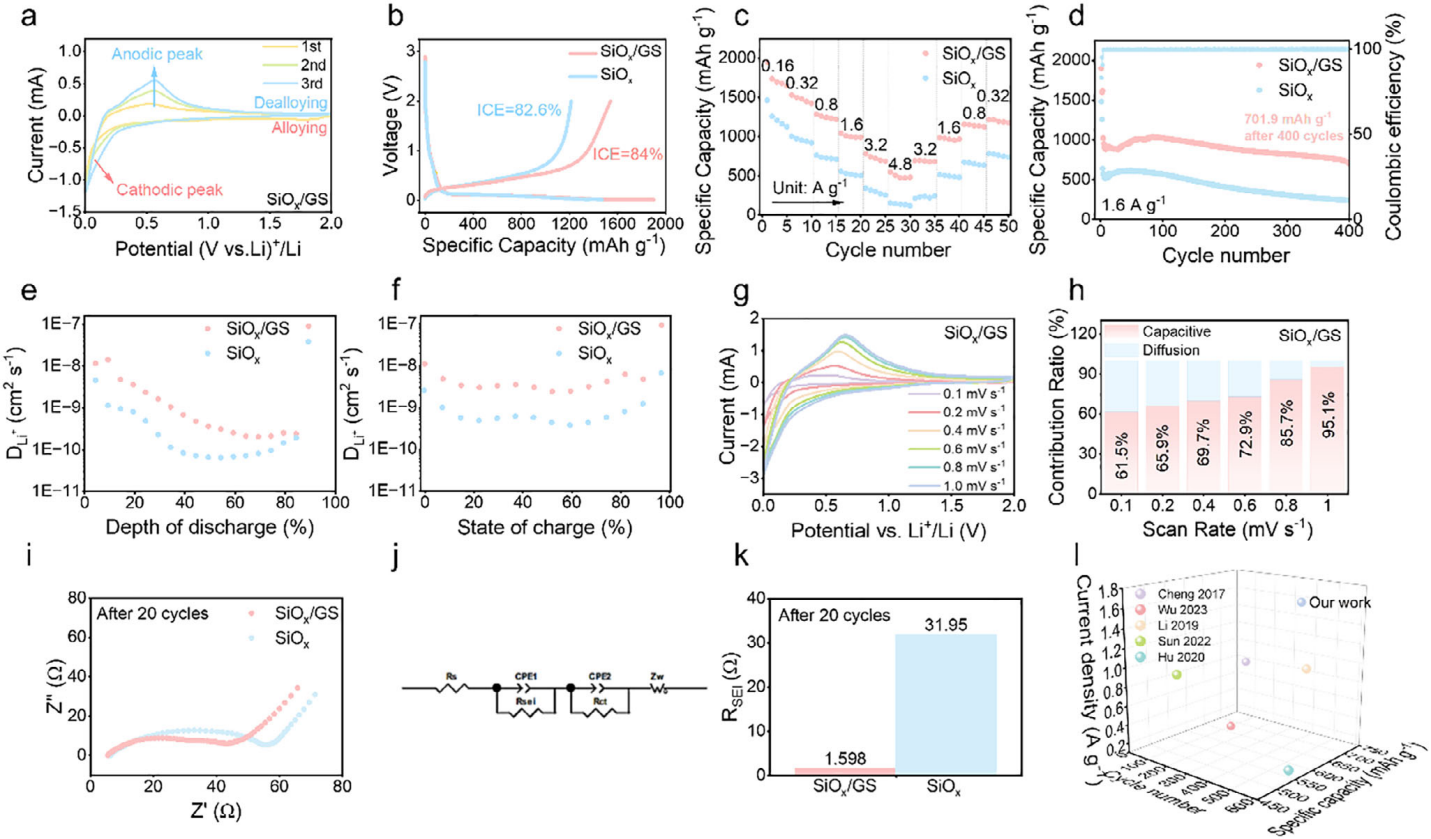
Electrochemical performance and kinetic analysis of the SiO_x_/GS electrode. (a) CV curves of the SiO_x_/GS electrode at 0.2 mV s^−1^. (b) First‐cycle galvanostatic charge/discharge profiles of the SiO_x_/GS and SiO_x_ electrodes. (c) Rate performance of the SiO_x_/GS and SiO_x_ electrodes at different current densities. (d) Cycling performance of the SiO_x_/GS and SiO_x_ electrodes at 1.6 A g^−1^. (e, f) GITT‐derived apparent electrode‐level Li‐ion transport coefficients (D__app_) of the SiO_x_/GS and SiO_x_ electrodes during e) discharge and f) charge processes. (g) CV curves of the SiO_x_/GS electrode at 0.1−1.0 mV s^−1^. (h) Capacitive and diffusion‐controlled current contributions of the SiO_x_/GS electrode at different scan rates. (i) Nyquist plots of the SiO_x_/GS and SiO_x_ electrodes after 20 cycles. (j) Equivalent circuit model used for EIS fitting. (k) Comparison of fitted SEI resistance (R_SEI_) values after 20 cycles. (l) Three‐dimensional comparison of the SiO_x_/GS electrode with recently reported SiO_x_‐based anodes in terms of specific capacity, cycle number, and current density [45, 46, 47, 48, 49, 50].

The first‐cycle galvanostatic charge/discharge profiles are shown in Figure 3b. The SiO_x_/GS electrode delivered an initial Coulombic efficiency of 84.0%, compared with 82.6% for pristine SiO_x_ [42]. In the rate test, SiO_x_/GS retained 507 mAh g^−1^ at 4.8 A g^−1^, whereas the capacity of pristine SiO_x_ was close to zero at the same current density (Figure 3c). After 400 cycles at 1.6 A g^−1^, the SiO_x_/GS electrode retained approximately 702 mAh g^−1^ (Figure 3d). A GS‐only half‐cell was used to assess the direct capacity contribution of the additive. At cycle 53, after three activation cycles at 0.2 A g^−1^ and 50 cycles at 2 A g^−1^, it retained a reversible delithiation capacity of 267.2 mAh g^−1^ (Figure S17). Scaling this value by the 4/70 GS‐to‐SiO_x_ mass ratio gives an estimated contribution of approximately 15.3 mAh per gram of SiO_x_ mass. This small contribution indicates that the improved performance mainly reflects preservation of SiO_x_ utilization through stress accommodation, electronic contact, and electrolyte accessibility.

GITT measurements were performed using 0.16 A g^−1^ pulses for 30 min followed by 3 h of relaxation (Figure S18). Under the same protocol, the SiO_x_/GS electrode showed smaller voltage transitions and faster relaxation than pristine SiO_x_. Because these measurements include composite‐electrode transport and polarization, the D_app_ values in Figure 3e,f are interpreted as apparent electrode‐level Li‐ion transport coefficients [43]. CV measurements for kinetic analysis were collected at scan rates from 0.1 to 1.0 mV s^−1^ (Figure 3g). The relation i = av^b^ was used to obtain b‐values, and i = k_1_v + k_2_v^1/2^ was used to separate fitted capacitive and diffusion‐controlled contributions [44]. For SiO_x_/GS, the capacitive contribution increased from 61.5% at 0.1 mV s^−1^ to 95.1% at 1.0 mV s^−1^ (Figure 3h and Figure S19), compared with 75% for pristine SiO_x_ at 1.0 mV s^−1^ (Figure S20). These results indicate a larger fitted surface‐controlled contribution in the GS‐containing electrode over the tested scan‐rate range.

EIS was used to compare interfacial and charge‐transfer resistances. After 20 activation cycles, the fitted SEI resistance (R_SEI_) was 1.60 Ω for SiO_x_/GS and 31.95 Ω for pristine SiO_x_ (Figure 3i–k and Figure S21). Spectra collected in the fresh state and after 10, 20, 50, and 100 cycles also showed smaller fitted solution and charge‐transfer resistances for SiO_x_/GS under the same measurement conditions (Figures S22 and S23). These data are consistent with lower interfacial resistance in the GS‐containing electrode. Compared with the cited SiO_x_ reports, the SiO_x_/GS electrode retained approximately 702 mAh g^−1^ after 400 cycles at 1.6 A g^−1^ (Figure 3l) [45, 46, 47, 48, 49, 50].

### 2.3 Working Mechanism of GS

Electrode morphology was examined at progressive lithiation depths. Cracks developed in pristine SiO_x_ during lithiation (Figure S24Information), whereas the optimized SiO_x_/GS electrode retained a more continuous surface (Figure S25). Cross‐sectional SEM provided a through‐plane measurement of the electrode response. Upon full lithiation, the SiO_x_/GS electrode thickness increased from 22.2 to 25.5 µm, corresponding to a 15% increase (Figure 4a,b), whereas the thickness of pristine SiO_x_ increased from 20.0 to 38.7 µm, corresponding to a 94% increase (Figure 4c,d and Figure S26). The lithiation‐depth‐dependent series is shown in Figures S27 and S28. These values describe through‐plane electrode‐thickness changes rather than three‐dimensional volume expansion.

**FIGURE 4:**
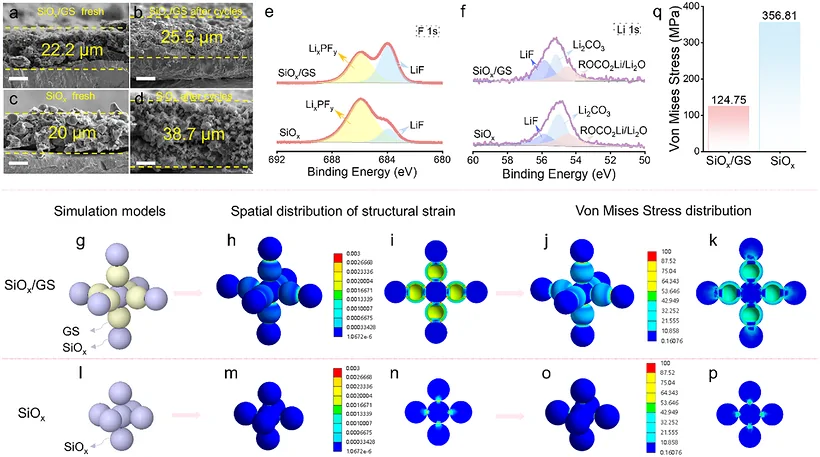
(a–d) Cross‐sectional images of the optimized SiO_x_/GS electrode (4 wt,% GS) and pristine SiO_x_ before and after full lithiation. (e, f) F 1s and Li 1s XPS spectra of SiO_x_/GS and SiO_x_ after 100 cycles at 0.32 A g^−1^. (g, l) Idealized FEM configurations. (h, i, m, n) Calculated strain distributions. (j, k, o, p) Calculated von Mises stress distributions. (q) Maximum von Mises stress under the same prescribed reference load. The FEM values compare two idealized model configurations and do not represent calibrated lithiation stresses. Panel q reports deterministic model outputs, not replicate means.

After 100 cycles at 0.32 A g^−1^, XPS showed a higher relative LiF contribution in the SiO_x_/GS electrode than in pristine SiO_x_ under the same cycling conditions (Figure 4e,f). AFM of the cycled surfaces showed a stiffer near‐surface response for SiO_x_/GS, and the corresponding SEM image showed fewer large cracks (Figures S30 and S31). XPS spectra were also collected from an aged GS‐only electrode after 53 physical cycles: three activation cycles at 0.2 A g^−1^ followed by 50 cycles at 2 A g^−1^ (Figure S29). LiF‐related components were present in the F 1s and Li 1s spectra, showing that a LiF‐containing interphase can form on GS in the FEC/LiPF_6_ electrolyte. Because the cycling histories differed, this result is discussed separately and is not used to assign the higher LiF contribution exclusively to GS.

Finite‐element analysis was used to compare stress‐localization trends in two idealized configurations under the same prescribed loading [51, 52]. The three‐dimensional static model represents SiO_x_ and the GS framework as isotropic, linear‐elastic homogeneous spheres. One central sphere was fixed, and six peripheral spheres applied the same inward reference load in both configurations; the material inputs are listed in Table S5 [53, 54, 55, 56]. The 20 GPa GS value represents the graphitic framework rather than the AFM‐derived apparent modulus of the complete hollow particle. Under these shared assumptions, the GS‐containing arrangement showed a lower maximum von Mises stress (approximately 125 MPa) than the pristine SiO_x_ arrangement (approximately 357 MPa; Figure 4g–q) [57]. The absolute values are comparative model outputs and are not interpreted as actual lithiation stresses.

### 2.4 Versatility Verification of GS

GS was also evaluated in SiO_x_/graphite (SiO_x_/G), Si@C [58, 59], and NCM811 formulations [60, 61, 62, 63]. In the SiO_x_/G test, SiO_x_/graphite/GS (SiO_x_/G/GS) showed an ICE of 86% and retained approximately 370 mAh g^−1^ after 300 cycles at 0.8 A g^−1^ (Figure S32). Screening of Si@C formulations identified 4 wt,% GS as the tested optimum (Figures S33–S37); the corresponding first‐cycle, cycling, and rate data are shown in Figure 5a–c. NCM811 was included as a cathode formulation that also undergoes structural change during cycling [64]. Here, 0.1 wt,% GS gave better retention than the tested 0.5 wt,% formulation (Figures S38–S42). NCM811/GS showed an ICE of 86% and retained 67% of its capacity after 155 cycles at 0.2 A g^−1^ (Figure 5d–f). A SiO_x_/GS||NCM811/GS full cell used a prelithiated anode and a nominal design target of N/P ≈ 1.1 [65]. The full‐cell active‐material masses and N/P calculations are provided in the Supporting Information. The full cell was cycled from 2.8 to 4.3 V at 0.5 C (1 C = 200 mA g^−1^ of NCM811), with capacity normalized to the NCM811 active mass. The GS‐containing full cell retained approximately 114 mAh g^−1^ after 200 cycles with an average Coulombic efficiency of 99.7% (Figure 5g,h).

**FIGURE 5:**
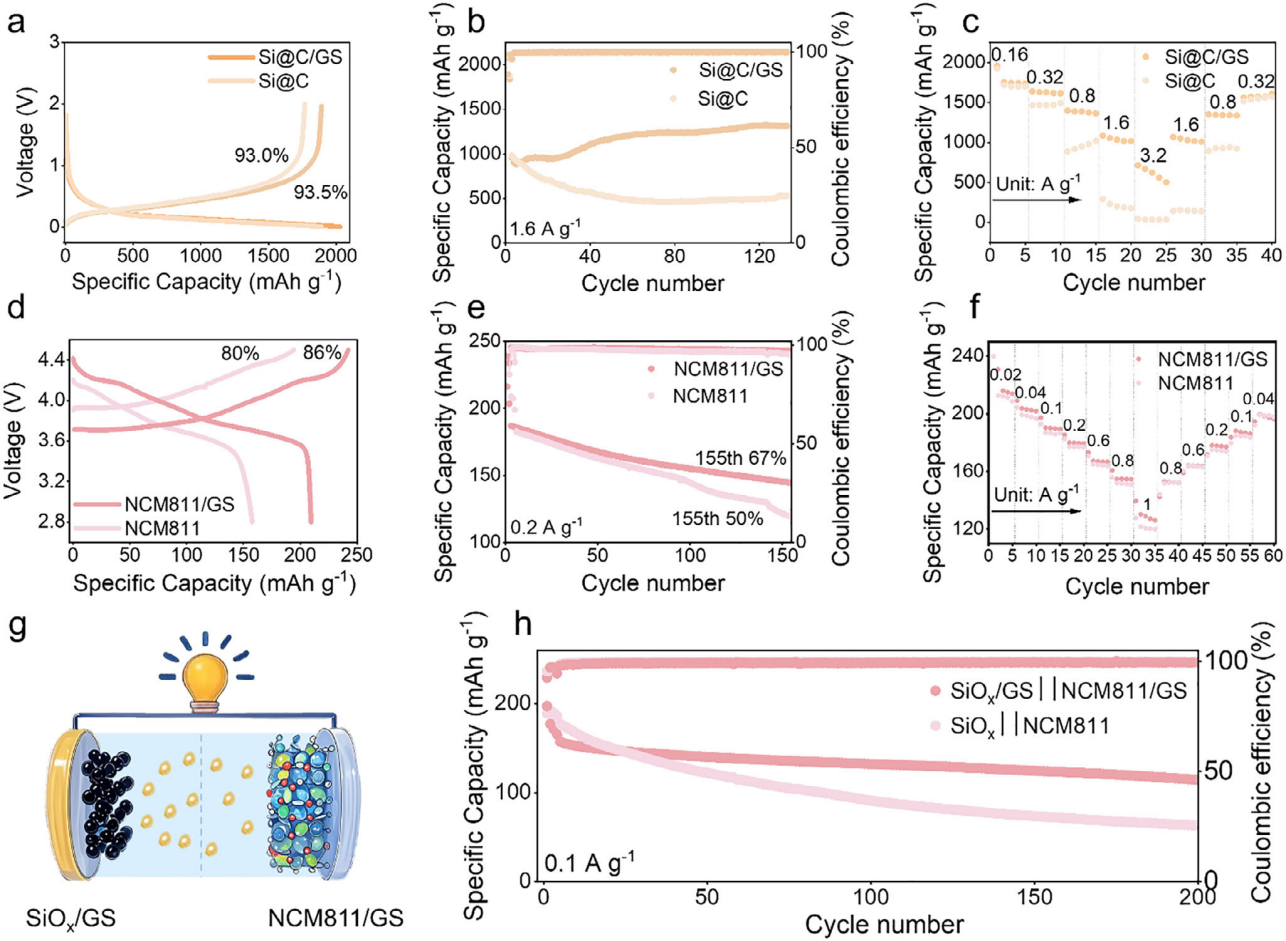
Performance of GS in the tested electrode systems. (a–c) First‐cycle profiles, cycling performance, and rate capability of Si@C/GS and Si@C electrodes. (d–f) First‐cycle profiles, cycling performance, and rate capability of NCM811/GS and NCM811 electrodes. (g) Schematic of the SiO_x_/GS||NCM811/GS full cell. (h) Cycling performance of the full cells; capacity is normalized to the NCM811 active mass.

Figure 6 integrates the experimental observations into a schematic interpretation of the coupled roles of GS. During lithiation, SiO_x_ expansion may compress neighboring hollow GS particles and utilize both GS cavities and pre‐existing electrode porosity; partial recovery during delithiation may then help preserve local separation and electronic contact. This proposed spring‐like response is based on the ex‐situ mechanical and morphological evidence described above. The conductive shell and porous architecture may also assist with electron transport and electrolyte access. Under the matched 100‐cycle protocol, XPS showed a higher relative LiF contribution in SiO_x_/GS than in pristine SiO_x_ (Figure 4e,f) [66, 67]. Taken together, the mechanical, transport, and interfacial observations support a multifunctional contribution of GS rather than a single causal pathway [68, 69].

**FIGURE 6:**
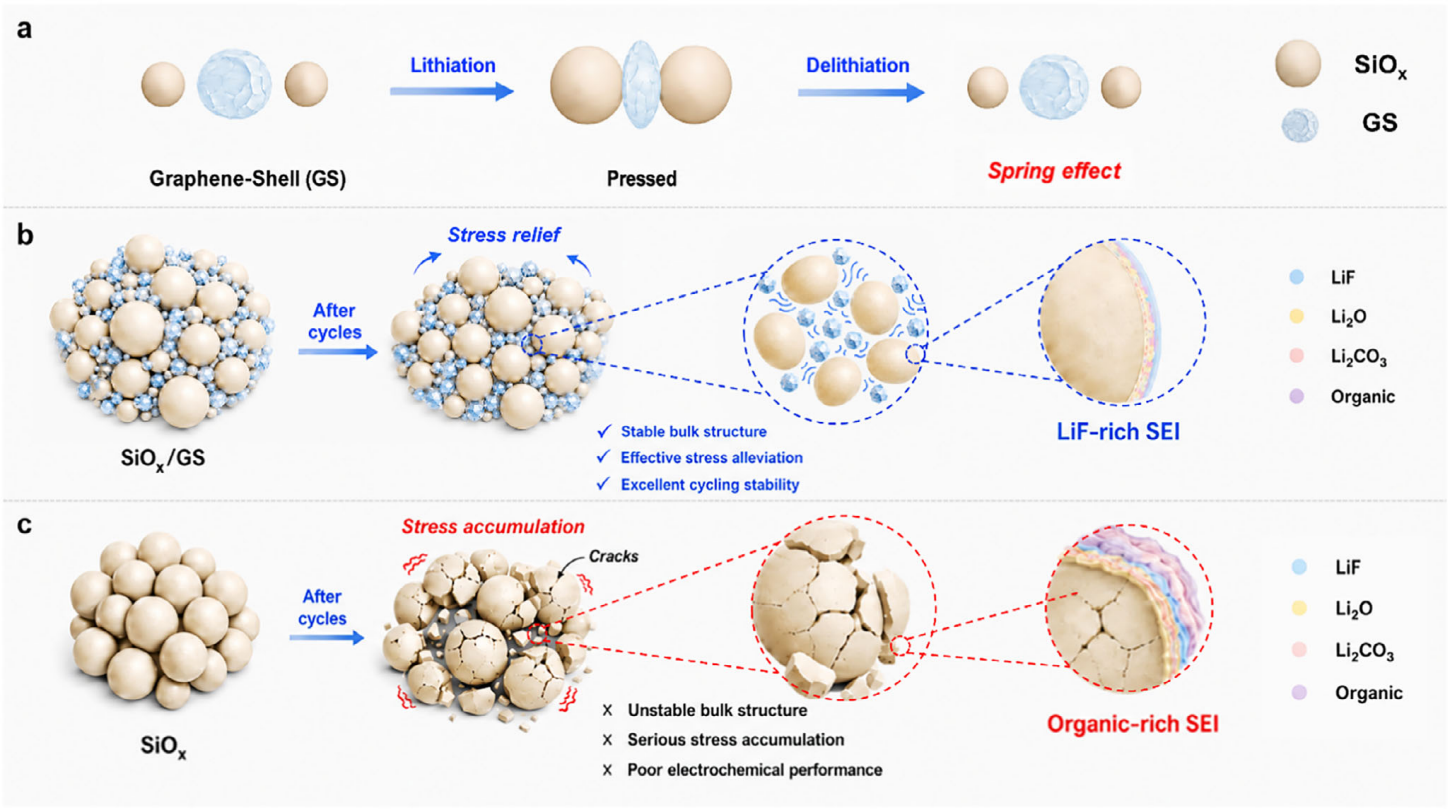
Schematic interpretation of coupled GS effects in SiO_x_‐based electrodes. a) Proposed local stress‐accommodation process during lithiation and delithiation; the depicted spring‐like motion is schematic. b) GS‐containing electrode, showing preserved bulk connectivity and the higher relative LiF contribution observed after cycling. c) Pristine SiO_x_ electrode, showing crack development and interfacial degradation.

## 3 Conclusion

Hollow graphene‐shell spheres were developed as slurry‐compatible pore‐engineering additives. In the tested SiO_x_ electrode, GS incorporation produced a smaller through‐plane thickness increase, greater recovery during the pressure cycle, lower fitted interfacial resistance, and improved capacity retention. The SiO_x_/GS electrode retained approximately 702 mAh g^−1^ after 400 cycles at 1.6 A g^−1^. Improvements were also observed in the tested SiO_x_/G, Si@C, and NCM811 electrodes. These results demonstrate a simple stress‐accommodating additive strategy for the electrode systems examined here.

## 4 Experimental Section

### 4.1 Materials

Lithiated poly(acrylic acid) (PAA‐Li solution, 6 wt,%), super P, graphite, and NCM811 powder were purchased from Canrd, China. Carboxymethylcellulose (CMC) was purchased from Aladdin, China. Spherical SiO_2_ (1 µm) was obtained from Shanghai Buwei Applied Materials Co., Ltd. Acetonitrile (electronic grade), polyvinylidene fluoride (PVDF, HSV900), and *N*‐methyl‐2‐pyrrolidone (NMP, electronic grade) were supplied by Shanghai Aladdin Biochemical Technology Co., Ltd. SiO_x_ and Si@C materials were provided by BTR New Material Group Co., Ltd. CNTs (diameter 4–6 nm, length ∼10 µm) were purchased from XFNANO, China.

### 4.2 Preparation of GS

GS was synthesized via CVD using 1 µm SiO_2_ spheres as the template. In a typical procedure, 4 g of SiO_2_ powder was weighed and placed in a quartz boat and then sealed within the CVD system. Graphitic carbon growth was carried out at 1000°C for 2 h with a heating rate of 20°C min^−1^. Pure acetonitrile was used as the carbon source and supplied to the CVD system via Ar gas flow at a rate of 300 mL min^−1^. The obtained product was washed with 5 mol L^−1^ NaOH three times, filtered using a vacuum filter, and then freeze‐dried under vacuum. The final product was stored in a centrifuge tube for future use. Further experimental details can be found in the Supporting Information.

### 4.3 Statistical Analysis

Electrochemical curves show data from individual cells rather than averages across cells. Particle‐size distributions were analyzed using Nano Measure. AFM modulus and dissipation histograms and map means describe spatial variation within the measured maps. No inferential statistical tests, p‐values, or significance symbols were used.

## Author Contributions

**Luwen Li, Qitao Shi**, and **Zhipeng Wang**: investigation, methodology, data curation, writing – original draft. **Cheng Zhang**, **Jiaqi Wang**, **Xiaoqin Yang**, and **Alicja Bachmatiuk**: formal analysis, visualization, validation. **Chen Lu**, **Ruizhi Yang**, **Yanbin Shen**, and **Mark H. Rümmeli**: conceptualization, supervision, project administration, funding acquisition, writing – review & editing.

## Conflicts of Interest

The authors declare no conflicts of interest.

## Supporting information




**Supporting File**: advs78085‐sup‐0001‐SuppMat.docx.

## Data Availability

The data that support the findings of this study are available on request from the corresponding author. The data are not publicly available due to privacy or ethical restrictions.
