# Supplementary material for: Elastic Graphene‐Shell Architectures Enable Stress Regulation in High‐Capacity Lithium‐Ion Battery Electrodes
Source: Adv Sci (Weinh). 2026 Oct 20:e78085. Online ahead of print. doi: 10.1002/advs.78085 (PMC13683979; doi:10.1002/advs.78085)
Supplement: Supplementary file 1 [file ADVS-9999-e78085-s001.docx]

**Supporting Information**

**Elastic Graphene-Shell Architectures Enable Stress Regulation in High-Capacity Lithium-Ion Battery Electrodes**

*Luwen Li^1^, Qitao Shi^1, *^, Zhipeng Wang^1^, Cheng Zhang^1^, Jiaqi Wang^1^, Xiaoqin Yang^2^, Alicja Bachmatiuk^3,4, *^, Chen Lu^5,*^, Ruizhi Yang^1,^*, Yanbin Shen^6,^*, and Mark H. Rümmeli^4,7,8,^**

^1^ College of Energy, Soochow Institute for Energy and Materials Innovations (SIEMIS), Key Laboratory of Advanced Carbon Materials and Wearable Energy Technologies of Jiangsu Province, Soochow University, Suzhou, P. R. China

^2^ Suzhou Laboratory, Suzhou 215123, China

^3^ Faculty of Chemistry, Wrocław University of Science and Technology, Wybrzeże Wyspiańskiego 27, 50-370 Wrocław, Poland

^4^ Electron Beam Emergent Additive Manufacturing (EBEAM) Centre, Centre for Nanotechnology (CNT), Centre for Energy and Environmental Technologies (CEET),

VSB—Technical University of Ostrava, 17. Listopadu 15, 708 33 Ostrava, Czech Republic

^5^ School of Electronic and Information Engineering, Changshu Institute of Technology, Changshu 215500, China

^6^ i-Lab, CAS Center for Excellence in Nanoscience, Suzhou Institute of Nano-Tech and Nano-Bionics (SINANO), Chinese Academy of Sciences (CAS), Suzhou 215123, P.R. China

^7^ Institute for Materials Chemistry, IFW Dresden, 20 Helmholtz Strasse, Dresden 01069, Germany

^8^ School of Smart Materials and Future Energy, Anhui Normal University,

Wuhu 214002, China

Correspondence: qtshi@suda.edu.cn (Q. Shi) | alicja.bachmatiuk@vsb.cz (A. Bachmatiuk

) | chenlu@cslg.edu.cn (Chen Lu) | yangrz@suda.edu.cn (Ruizhi Yang) | ybshen2017@sinano.ac.cn (Yanbin Shen) | mhr1@vsb.cz (M. H. Rümmeli)

Experimental Section

Material characterizations

*XRD measurement:* Phase characterization was performed by X-ray diffraction (XRD, Bruker D8 Advance) at 2θ angles between 10° and 80° using Cu Kα radiation (*λ* = 1.5406 Å).

*Raman measurement:* Raman spectra were acquired with a confocal Raman spectrometer (HR Evolution) using a 633 nm laser. For Raman measurements, aged batteries were disassembled in a glove box to extract the electrodes. The electrodes were rinsed three times with dimethyl carbonate (DMC, AR, 99%, Aladdin) and then dried in a vacuum oven for 2 h. The obtained electrodes were used directly for Raman measurements.

*SEM measurement:* The powder and electrode samples were directly mounted onto the scanning electron microscopy (SEM) specimen stage using conductive tape. Morphologies and energy-dispersive X-ray spectroscopy (EDS) maps were obtained using a scanning electron microscope (SU8010, Hitachi) operated at 10 kV and 10 mA. Particle sizes were measured from low-magnification SEM images and analyzed using Nano Measure software.

*TEM measurement:* The powder was dispersed in ethanol and sonicated to achieve a uniform dispersion. A 20 μL aliquot of the dispersion was dropped onto a Cu grid and dried at 60 °C. Transmission electron microscopy (TEM) images were obtained using a spherical aberration-corrected FEI Titan Themis Cubed G2 300 TEM operated at an accelerating voltage of 300 kV.

*AFM measurement: Atomic force microscopy measurements were performed using a Bruker Dimension ICON system equipped with an RTESPA-525 probe. For Fig. 2c, force spectroscopy was performed on an individual GS particle, and an apparent whole-particle modulus was obtained using a Hertz-model fit within the recorded small-displacement range. Fresh and cycled electrode surfaces were measured separately; cycled electrodes were removed from cells, rinsed with DMC, and dried before analysis. The archived method record does not contain the calibrated tip radius, Poisson’s ratio, maximum force, fit-window limits, map area, pixel count, or number of independently sampled regions; these values were therefore not inferred.*

*XPS measurement of the GS-only electrode:* The aged GS-only electrode was recovered after three activation cycles at 0.2 A g⁻¹ followed by 50 cycles at 2 A g⁻¹ (53 physical cycles in total). The electrode was handled and analyzed using the same ex-situ procedure and spectral-fitting method as the other cycled electrodes.

*Pressure-dependent conductivity measurement:* GS powder and electrode conductivity were measured using a PRCD3100 powder resistivity and compaction-density analyzer (IEST, China). Resistance and sample thickness were recorded under applied pressure, and conductivity was calculated as σ = L/(RA), where L, R, and A are the sample thickness, resistance, and contact area, respectively. GS powder was tested up to 200 MPa, whereas the electrode samples were tested from 5 to 60 MPa.

*Contact-angle and electrolyte-retention measurements:* The electrolyte was 1.0 M LiPF₆ in EC/DEC (1:1 by volume) containing 10 wt% FEC and 1 wt% VC. Equal-volume droplets were placed on freshly prepared electrode surfaces for the contact-angle measurements. For electrolyte retention, the dry electrode mass (m₀) and the mass after soaking followed by the same drain/blot procedure (m₁) were recorded, and retention was calculated as (m₁ − m₀)/m₀.

*Electrode preparation:* The detailed formulations of all electrode compositions are summarized in Table S1. Taking the SiO_x_/GS electrode as an example, SiO_x_, Super P, and GS powder were first mixed by hand milling in a mortar. These powders were then mixed with SA binder and SWCNTs in a vial by magnetic stirring for at least 5 h to obtain a slurry. This slurry was coated onto the Cu foil using a doctor blade and dried at 60 °C to remove moisture. The as-prepared electrode was cut into 14 mm discs, followed by vacuum drying at 80 °C overnight; full-cell anodes and cathodes used the diameters specified below. Other electrodes were prepared using similar steps. Unless otherwise stated, SiO_x_/GS in the mechanical, electrochemical, and lithiation-depth measurements denotes the optimized 4 wt% GS formulation. The 20 wt% GS electrode in Fig. 2a was prepared only for cross-sectional visualization of the hollow particles. The active-material areal loadings of the SiOₓ/GS, SiOₓ, Si@C/GS, and Si@C half-cell electrodes were approximately 0.7–0.9 mg cm⁻². For the GS-only control, GS was used as the active material in the same CR2032 half-cell configuration and with the same FEC/LiPF₆-containing electrolyte as the SiOₓ-based half-cells. Specific capacity and current density were normalized to the mass of GS.

*Battery assembly:* The performance of the working electrode was evaluated by assembling CR2032 coin cells. Lithium foil was used as the counter electrode, and a polypropylene membrane was used as the separator. The electrolyte was 1 mol L^–1^ lithium hexafluorophosphate (LiPF_6_) dissolved in diethyl carbonate (DEC) and ethylene carbonate (EC) (1:1 by volume) with 10 wt% fluoroethylene carbonate (FEC) and 1 wt% vinylene carbonate (VC) as additives. Each cell contained 80 μL of electrolyte per cell.

*Full-cell assembly:* SiOₓ/GS||NCM811/GS and SiOₓ||NCM811 coin cells were assembled in an Ar-filled glove box using 1.0 M LiPF₆ in EC/DEC/DMC (1:1:1 by volume) and a Celgard-2400 separator. Both anodes were prelithiated in half-cells for three cycles at 0.1 A g⁻¹. The anode and cathode diameters were 16 and 14 mm, respectively. For the individual full cells presented here, the SiOₓ and NCM811 active-material masses were 1.190 and 8.608 mg in the GS-containing cell and 1.162 and 8.416 mg in the GS-free control, respectively. Active-material masses were obtained by subtracting the current-collector masses and applying the active-material fractions in Table S2. The whole-disc N/P ratios were 1.106 and 1.105, respectively, calculated as (m_N_q_N_)/(m_P_q_P_) using balancing specific capacities of 1600 mAh g⁻¹ for SiOₓ and 200 mAh g⁻¹ for NCM811. Full cells were cycled from 2.8 to 4.3 V at 0.5 C (1 C = 200 mA g⁻¹ of NCM811), and capacity was normalized to the NCM811 active mass. Three independently assembled SiOₓ/GS||NCM811/GS full cells were tested under the same cycling conditions (n = 3).

*Electrochemical tests:* The electrochemical performance of SiO_x_-based electrodes was investigated using a LAND battery testing system by galvanostatic charge–discharge measurements. The batteries were activated at 0.1 C for three cycles and tested at 0.2 or 1 C to evaluate long-term cycling performance. To evaluate rate capability, the batteries were tested at current densities of 0.1, 0.2, 0.5, 1, 2, and 3 C for five cycles each. Electrochemical impedance spectra (EIS) were obtained at frequencies ranging from 100 kHz to 0.01 Hz with a bias voltage of 0.005 V. The initial-cycle CV curves in Fig. 3a and Fig. S16 were collected at 0.2 mV s^–1^, whereas the kinetic analysis in Fig. 3g and Figs. S19–S20 used scan rates of 0.1, 0.2, 0.4, 0.6, 0.8, and 1.0 mV s^–1^ on a CHI600E electrochemical workstation. For calendar aging, the batteries assembled inside the glove box were extracted and stored in a valve bag at room temperature (approximately 26 ℃) for three days before EIS measurements. To conduct ex-situ EIS measurements, the battery was discharged or charged to a certain voltage and then tested using an electrochemical workstation. Galvanostatic intermittent titration technique (GITT) measurements were conducted on a Neware battery testing system between 0.005 and 2 V with a constant pulse current of 0.16 A g^-1^ for 30 min, followed by 3 h relaxation intervals. The GS-only half-cell was activated at 0.2 A g⁻¹ for three cycles and subsequently cycled at 2 A g⁻¹ for 50 cycles.

*GITT analysis:* The apparent electrode-level Li-ion transport coefficient was calculated using

$$D_{\text{app}}=\frac{4}{\pi\tau}\left( \frac{m_{\text{B}}V_{\text{M}}}{M_{\text{B}}A} \right)^{2}\left( \frac{\Delta E_{\text{s}}}{\Delta E_{\tau}} \right)^{2}$$

, where τ is the pulse duration, mB is the active-material mass, VM and MB are the molar volume and molar mass, respectively, A is the electrode area, ΔE_τ_ is the voltage change during the current pulse after removal of the instantaneous iR drop, and ΔE_s_ is the steady-state voltage change between adjacent relaxation steps. Because the two electrodes differ in thickness, porosity, and interfacial resistance, the calculated D_app_ values are treated as comparative electrode-level transport parameters rather than intrinsic solid-state diffusion coefficients. The archived analysis record does not preserve the numerical V_M_, M_B_, m_B_, and A inputs used for each calculation; absolute D_app_ values therefore cannot be independently reconstructed from the present package, and the direct voltage-transition and relaxation comparison is emphasized.

Finite element simulations

A three-dimensional static structural model was used to compare idealized spherical SiOₓ/GS and SiOₓ arrangements. Both materials were treated as isotropic, linear-elastic media. The central sphere was fixed, and six surrounding spheres applied the same inward prescribed load. The 500 N force was used only as an arbitrary common reference and does not represent the actual force on a micrometer-scale particle. GS was represented as a homogeneous effective sphere rather than an explicit hollow shell. Tetrahedral elements and the APDL solver were used. The model does not resolve the explicit cavity, shell buckling, friction, evolving contact, or lithiation eigenstrain. The archived model record available for revision does not include geometric dimensions, element counts, mesh-convergence results, load-application area, or the large-deformation setting; no values were inferred. Calculated stresses are therefore reported only as comparative outputs under the stated model assumptions.

**Statistical analysis:** Electrochemical curves show data from individual cells rather than averages across cells. Particle-size distributions were analyzed using Nano Measure. AFM modulus and dissipation histograms and map means describe spatial variation within the measured maps. No inferential statistical tests, p-values, or significance symbols were used.

**Table S1.** Composition of GS-modified electrodes and their control counterparts

| Electrodes | Active Materials | | Binder | Conductive Additives | GS |
| --- | --- | --- | --- | --- | --- |
| SiO_x_/0 wt% GS | SiO_x_(70) | SA (10) | | SWCNTs (2)/Super P (18) | 0 |
| SiO_x_/2 CMC (5)/PAA-Li (5) wt % GS | SiO_x_(70) | SA (10) | | SWCNTs (2)/Super P (16) | 2 |
| SiO_x_/4 wt% GS | SiO_x_ (70) | SA (10) | | SWCNTs (2)/Super P (14) | 4 |
| SiO_x_/6 wt% GS | SiO_x_ (70) | SA (10) | | SWCNTs (2)/Super P (12) | 6 |
| Si@C/0 wt% GS | Si@C (75) | CMC (5)/PAA-Li (5) | | SWCNTs (1)/Super P (14) | 0 |
| Si@C/2 wt% GS | Si@C (75) | CMC (5)/PAA-Li (5) | | SWCNTs (1)/Super P (12) | 2 |
| Si@C/4 wt% GS | Si@C (75) | CMC (5)/PAA-Li (5) | | SWCNTs (1)/Super P (10) | 4 |
| Si@C/6 wt% GS | Si@C (75) | CMC (5)/PAA-Li (5) | | SWCNTs (1)/Super P (8) | 6 |
| NCM811/0 wt% GS | NCM (80) | PVDF (10) | | Super P (10) | 0 |
| NCM811/0.1 wt% GS | NCM (80) | PVDF (10) | | Super P (9.9) | 0.1 |
| NCM811/0.5 wt% GS | NCM (80) | PVDF (10) | | Super P (9.5) | 0.5 |

**Table S2.** Optimized GS-modified electrodes and their corresponding control samples used for performance comparison.

| Electrodes | Active Materials | Binder | | Conductive Additives | GS |
| --- | --- | --- | --- | --- | --- |
| SiO_x_/GS | SiO_x_(70) | | SA (10) | SWCNTs (2)/Super P (14) | 4 |
| *SiO_x_ | SiO_x_(70) | | SA (10) | SWCNTs (2)/Super P (18) | - |
| SiO_x_/G/GS | SiO_x_/G (70) | | SA (10) | SWCNTs (2)/Super P (14) | 4 |
| *SiO_x_/G | SiO_x_/G (70) | | SA (10) | SWCNTs (2)/Super P (18) | - |
| Si@C/GS | Si@C (75) | | CMC(5)/PAA-Li (5) | SWCNTs (1)/Super P (10) | 4 |
| *Si@C | Si@C (75) | | CMC(5)/PAA-Li (5) | SWCNTs (1)/Super P (14) | - |
| NCM811/GS | NCM (80) | | PVDF (10) | Super P (9.9) | 0.1 |
| *NCM811 | NCM (80) | | PVDF (10) | Super P (10) | - |

*Notes: All values are presented in weight percent (wt%). GS: graphene-shell spheres; SA: sodium alginate; CMC: carboxymethyl cellulose; PAA-Li: poly(acrylic acid) lithium salt; NCM811: LiNi_0.8_Co_0.1_Mn_0.1_O_2_;* *electrodes marked with an asterisk represent control samples.*

**Fig. S1** Disadvantage of the chemical etching method.

**Fig. S2** Disadvantage of the thermal treatment method.

**Fig. S3** Introduction of pore-forming agents

**Fig. S4** Schematic of GS preparation.

**Fig. S5** Particle size distribution of GS.

**Fig. S6** XRD pattern of GS.

**Fig. S7** Raman spectrum of GS.

**Fig. S8** N₂ adsorption–desorption isotherm of GS.

**Fig. S9** Comparison of cycling performance of SiO_x_ electrodes with different GS contents (0, 2, 4, 6 wt%)

**Fig. S10** Surface SEM images of SiO_x_ electrodes with different GS contents before (fresh) and after cycling: **a, b)** 2 wt% GS; **c, d)** 4 wt% GS; **e, f)** 6 wt% GS.

**Fig. S11** Nyquist plots of SiO_x_ electrodes with different GS contents, measured in the fresh state and after 50 cycles: **a)** 2 wt% GS, **b)** 4 wt% GS, and **c)** 6 wt% GS.

**Fig. S12** **a)** SEI-derived (*R*_SEI_) and **b)** charge-transfer resistances (*R*_ct_) after 50 cycles for SiO_x_ electrodes with different GS contents.

**Table S3 Original thickness data recorded during the 5–60–5 MPa loading–unloading test.**

| Pressure (MPa) | SiOₓ/GS electrode thickness (µm) | Percentage change in SiO_x_/GS electrode thickness | SiOₓ electrode thickness (µm) | Percentage change in SiO_x_ electrode thickness |
| --- | --- | --- | --- | --- |
| 5 | 28.7 | 0.00% | 24.2 | 0.00% |
| 10 | 27.1 | 5.57% | 22.8 | 5.79% |
| 15 | 26.2 | 8.71% | 22.1 | 8.68% |
| 20 | 25.6 | 10.80% | 21.5 | 11.16% |
| 25 | 24.8 | 13.59% | 20.9 | 13.64% |
| 30 | 24.4 | 14.98% | 20.6 | 14.88% |
| 35 | 23.9 | 16.72% | 20.1 | 16.94% |
| 40 | 23.5 | 18.12% | 19.8 | 18.18% |
| 45 | 23.0 | 19.86% | 19.4 | 19.83% |
| 50 | 22.8 | 20.56% | 19.1 | 21.07% |
| 55 | 22.5 | 21.60% | 18.9 | 21.90% |
| 60 | 22.3 | 22.30% | 18.8 | 22.31% |
| 55 | 22.0 | 23.34% | 18.4 | 23.97% |
| 50 | 21.9 | 23.69% | 18.3 | 24.38% |
| 45 | 21.9 | 23.69% | 18.3 | 24.38% |
| 40 | 22.0 | 23.34% | 18.3 | 24.38% |
| 35 | 22.0 | 23.34% | 18.3 | 24.38% |
| 30 | 22.2 | 22.65% | 18.4 | 23.97% |
| 25 | 22.1 | 23.00% | 18.3 | 24.38% |
| 20 | 22.3 | 22.30% | 18.4 | 23.97% |
| 15 | 22.4 | 21.95% | 18.4 | 23.97% |
| 10 | 22.5 | 21.60% | 18.4 | 23.97% |
| 5 | 23.1 | 19.51% | 18.6 | 23.14% |

**Table S4 Mechanical parameters extracted from the 5–60–5 MPa loading–unloading curves.**

| Sample | Maximum observed thickness variation (ε_max_) | Residual thickness variation at final 5 MPa (ε_final,5 MPa_) | Thickness-recovery index (I_rec_; percentage points) | Normalized recovery fraction, I_rec_/ε_max_ |
| --- | --- | --- | --- | --- |
| Pristine SiOₓ | 24.38% | 23.14% | 1.24 | 5.1% |
| SiOₓ/GS | 23.69% | 19.51% | 4.18 | 17.6% |

**Calculation of the Thickness-Recovery Index:** The pressure sequence was recorded from a 5 MPa seating load to 60 MPa and back to 5 MPa. The thickness-recovery index was calculated as I_rec_ = ε_max_ − ε_final,5 MPa_. Because ε is reported as a percentage thickness reduction, I_rec_ is a difference in percentage points. For SiOₓ/GS, I_rec_ = 23.69% − 19.51% = 4.18 percentage points; for pristine SiOₓ, I_rec_ = 24.38% − 23.14% = 1.24 percentage points. The corresponding normalized recovery fractions, I_rec_/ε_max_ × 100%, are 17.6% and 5.1%, respectively. The maximum reduction occurred shortly after pressure reversal, indicating time-dependent relaxation; the index is therefore a descriptive recovery metric for this pressure cycle and not an intrinsic elastic constant.

**Fig. S13** Pressure-dependent conductivity of the optimized SiO_x_/GS and pristine SiO_x_ electrodes.

**Fig. S14** Young’s modulus distributions: **a)** SiO_x_/GS. **b, c)** SiO_x_. The distributions describe pixel-level spatial variation in the displayed maps, not variation among independent electrodes.

**Fig. S15** Energy dissipation distributions: **a)** optimized SiO_x_/GS; **b, c)** pristine SiO_x_. The corresponding mean values are approximately 3.8 and 6.3 keV. These are pixel-level map means.

**Fig. S16** CV curves of pristine SiO_x_ collected at 0.2 mV s^−1^.

**Fig. S17** Electrochemical behavior of the GS-only electrode. **A)** Cycling performance and Coulombic efficiency. The first three cycles were collected at 0.2 A g⁻¹ for activation, followed by 50 cycles at 2 A g⁻¹. **b)** Galvanostatic voltage profiles for physical cycles 1, 2, 3, 4, 20, and 53. Specific capacity and current density were normalized to the mass of GS.

**Fig. S18** Time–voltage curve from the GITT test. Under the same pulse conditions, the SiO_x_/GS electrode exhibits a smaller voltage transition and faster relaxation than pristine SiO_x_; the derived D__app_ values are treated as apparent electrode-level transport parameters.

**Fig. S19** **a)** Log(i) versus log(v) plots of the cathodic and anodic peaks of SiO_x_/GS using scan rates of 0.1−1.0 mV s^−1^. **b)** CV curve and capacitive current contribution at 1.0 mV s^−1^.

**Fig. S20** **a)** CV curves of pristine SiO_x_ at 0.1, 0.2, 0.4, 0.6, 0.8, and 1.0 mV s^−1^. **b)** Log(i) versus log(v) plots of the cathodic and anodic peaks. **c)** Capacitive and diffusion contributions as a function of scan rate. **d)** Capacitive current contribution at 1.0 mV s^−1^.

**Fig. S21** Supplementary EIS analysis of the SiO_x_/GS and SiO_x_ electrodes after 20 cycles: **a)** Nyquist plots and **b)** fitted SEI resistance (R_SEI_) values.

**Fig. S22** Nyquist plots of the **a)** SiO_x_/GS and **b)** SiO_x_ electrodes in the fresh state and after different numbers of cycles.

**Fig. S23** Fitted resistance values of SiO_x_/GS and SiO_x_ electrodes at different cycling states: **a)** solution resistance (R_s_) and **b)** charge-transfer resistance (R_ct_).

**Fig. S24** **a–f)** Ex-situ top-view SEM images of pristine SiO_x_ at the initial state and discharge depths of 20%, 40%, 60%, 80%, and 100%. Scale bar: 5 μm.

**Fig. S25** **a–f)** Ex-situ top-view SEM images of the optimized SiO_x_/GS electrode (4 wt% GS) at the initial state and discharge depths of 20%, 40%, 60%, 80%, and 100%. Scale bar: 5 μm.

**Fig. S26** Thickness variation of the optimized SiO_x_/GS and pristine SiO_x_ electrodes after complete lithiation. These percentages describe the reported thickness comparison.

**Fig. S27** **a–f)** Ex-situ cross-sectional SEM images of the optimized SiO_x_/GS electrode (4 wt% GS) at the initial state and different discharge depths. Scale bar: 10 μm.

**Fig. S28** **a–f)** Ex-situ cross-sectional SEM images of pristine SiO_x_ at the initial state and different discharge depths. Scale bar: 10 μm.

**Fig. S29** XPS spectra of the aged GS-only electrode after three activation cycles at 0.2 A g⁻¹ followed by 50 cycles at 2 A g⁻¹ (53 physical cycles in total). **a**) F 1s and **b**) Li 1s spectra with fitted components. LiF-related components confirm the formation of a LiF-containing interphase on the cycled GS surface in the FEC/LiPF₆ electrolyte.

**Fig. S30** **a)** Surface SEM image of the optimized SiO_x_/GS electrode after 100 cycles. **b, c)** Young’s modulus map and distribution of the cycled electrode surface. The AFM distribution is pixel-level.

**Fig. S31** **a)** Surface SEM image of pristine SiO_x_ after 100 cycles. **b, c)** Young’s modulus map and distribution of the cycled electrode surface. The AFM distribution is pixel-level.

**Table S5** Simulation parameters of mechanical constants.

| **Parameters** | **Values** |
| --- | --- |
| Young’s modulus of SiO_x_ | 152.2 GPa |
| Young’s modulus of GS | 20 GPa |
| Poisson’s ratio of SiO_x_ | 0.21 |
| Poisson’s ratio of GS | 0.23 |

Note: The 20 GPa value used for GS represents the graphitic framework, whereas the AFM-fitted 114 MPa value is the apparent modulus of the complete hollow particle. The FEM uses a homogeneous effective sphere and is intended only for comparison between the two model configurations.

**Fig. S32** **a)** Capacity voltage curve of SiO_x_/G/GS and SiO_x_ in the first cycle. **b)** Cycling performance of SiO_x_/G/GS and SiO_x_/G at 0.8 A g^–1^. **c)** Rate performance of SiO_x_/G/GS and SiO_x_/G.

**Fig. S33** Cycling performance of Si@C electrodes with different GS contents at a current density of 1.6 A g^–1^

**Fig. S34** Cross-sectional SEM images of Si@C electrodes with different GS contents before (Fresh) and after cycling. **a, b)** 2 wt% GS, **c, d)** 4 wt% GS, and **e, f)** 6 wt% GS.

**Fig. S35** Top-view SEM images of Si@C electrodes with different GS contents before (Fresh) and after cycling. **a, b)** 2 wt% GS, **c, d)** 4 wt% GS and **e, f)** 6 wt% GS.

**Fig. S36** Nyquist plots of Si@C electrodes with different GS contents measured in the fresh state and after 50 cycles: **a)** 2 wt% GS, **b)** 4 wt% GS, and **c)** 6 wt% GS.

**Fig. S37** Resistances after 50 cycles for Si@C electrodes with different GS contents: **a)** *R*_SEI_ and **b)** charge-transfer resistance *R*_ct_.

**Fig. S38** Cycling performance of NCM811 electrode with different GS contents at a current density of 0.2 A g^–1^

**Fig. S39** Top-view SEM images of NCM811 electrodes with different GS contents before (Fresh) and after cycling. **a, b)** 0 wt% GS, **c, d)** 0.1 wt% GS and **e, f)** 0.5 wt% GS.

**Fig. S40** Cross-sectional SEM images of NCM811 electrodes with different GS contents before (Fresh) and after cycling. **a, b)** 0 wt% GS, **c, d)** 0.1 wt% GS, **e, f)** 0.5 wt% GS.

**Fig. S41** Nyquist plots of NCM811 electrodes with different GS contents measured in the fresh state and after 50 cycles: **a)** 0 wt% GS, **b)** 0.1 wt% GS, and **c)** 0.5 wt% GS.

**Fig. S42** SEI-derived (*R*_SEI_) and charge-transfer resistances (*R*_ct_) after 50 cycles for NCM811 electrode with different GS contents.
